# Waves of Extremism: An Applied Ethnographic Analysis of the Bosnia and Herzegovina Football Terraces

**DOI:** 10.3389/fspor.2022.770441

**Published:** 2022-03-29

**Authors:** Alberto Testa

**Affiliations:** School of Human and Social Sciences, University of West London, London, United Kingdom

**Keywords:** extremism, far-right, Bosnia, Balkans, football, violence, Ultras, policing

## Abstract

This article offers an overview of a four-month research project, conducted in 2019/2020, which studied extremism in the Bosnia and Herzegovina (BiH) football terraces. This work was funded by the International Organisation for Migration - United Nations and by the United States Agency for International Development (USAID). The research focused on risk factors and how these may govern the “entry” of BiH youth into extreme hard-core football fans groups (Ultras^1^) and prolong their involvement in them. The study highlighted the nature of these groups and their activity providing detailed recommendations for BiH policymakers, security agencies, and football federations and clubs who wish to understand and effectively respond to this emergent threat for public security in BiH.

## Introduction: The Bosnia and Herzegovina Social Space

As a result of the 1995 Dayton Agreement, Bosnia and Herzegovina (BiH) is today a country divided both geographically and politically along religious and ethnic lines, existing as a tripartite state. Within the population of 3.254 million, 48% identify as Bosniak (Bosnian Muslims), 37% as Bosnian Serbs, the majority of whom are Orthodox, and 14% as Bosnian Croats, most of whom are Catholic (World Population Review, 2020)^2^. A crucial factor in the politics, society, and international engagement of BiH is the impact of the ethnic and religious conflicts of the 1990s. The legacy of these conflicts continues to control the narratives that meld religion, heritage, culture, and ethnicity in the evolution of Bosnian identities, driving difference and division. For example, far-right “Chetnik” groups (named after Serbo-Croat units within the former Yugoslav Army) depend primarily on the Ravna Gora movement, centred mainly in Prijedor's north-western town^3^. The Neo-Ustaše groups^4^ (the former Croatian fascist movement) are especially active in areas along the BiH Croatian border where ethnic tensions flare-up between the predominantly Croatian population and the Bosniak Muslims. Bosnian Muslims also have nationalist or extremist groups. Nascent factions have appeared, including the Bosnian Movement of National Pride (BPNP). The movement promotes Bosniak identity and supports a secular Bosniak ethno-nationalist state; it actively shows enmity towards groups such as Roma, Communists, Jewish people, the LGBT community, and the so-called “non-whites” considered alien to their ideology and cultural heritage^5^.

Against this background, hostilities are frequently expressed across BiH football terraces, with clashes among groups of Ultras. For example, widespread fights involving over 500 fans took place in 2009 in the Bosnian Croat-dominated town of Široki Brijeg between locals and the Ultras from FK Zeljeznicar^6^ resulting in one death, over fifty injured, and widespread vandalism^7^. In 2015, the Široki Brijeg youth football team bus was ambushed in Sarajevo, once again involving Ultras connected to FK Zeljeznicar^8^. In other towns with diverse populations such as Mostar, sectarian divides also permeate football clubs' rivalries; for instance, FK Velež Mostar (associated with Bosniaks) and HŠK Zrinjski Mostar (linked to Bosnian Croats). Because of such violent episodes, the Bosnian Football Union has regularly ordered football matches to be played behind closed doors. Over the past decade, there have been repeated rumours that the national football league – currently consisting of Bosniak, Bosnian Serb, and Bosnian Croat teams – could be dissolved. On occasions, Bosnian Serb officials have suggested withdrawing Bosnian Serb football teams from the league altogether; the NK Široki Brijeg football club management has several times threatened to join Croatia's national football league instead^9^. Such violence highlights the fragility of tolerance between the country's three ethnic groups and how easily intolerance can escalate into violence, often stoked by far-right and nationalist groups.

Most studies on the link between extremism and violent ideologically Ultras groups rely on “external” observations of the groups' behaviours and mostly on secondary collected data from the Internet *via* social media^10^. This approach is understandable as security risks are involved in interacting with these groups, and these groups are challenging to approach^11^. This situation is even more difficult considering the peculiarity of the BiH social space, as mentioned earlier, plagued by historical conflicts among different ethnic groups and political rivalries among the same ethnic groups. Few studies have focused on this topic; among the most notable, Milojević et al. (2013) mainly link the occurrences of violence among Ultras to the consequences of the BiH war; however, it is dated. The excellent research of Italian sociologist Sterchele (2013) provides an ethnographic account of BiH football practitioners, including BiH football supporters, but not focusing specifically on the groups analysed by this article.

This article aims to fill this gap in the literature; the article originates from a research project aimed to understand the potential linkage between Ultras in BiH and violent extremism. This article will focus only on findings aiming to understand the BiH Ultras groups' main traits, exploring if they can be considered extreme football fans or, more simply, criminal gangs interested in football. This article will also explain who joins these groups, the groups' structure, their appeal to the BiH youth, and how the groups use social media to manifest their collective identity and, if any, ideologies.

## Methods

This study employed an “applied ethnographic” approach^12^. Applied ethnography has two main elements; the first one is explanatory, therefore, relevant for policymakers, practitioners, and institutions, and anyone seeking to address complex social issues. The second element is the application to real-world problems; it provides a specific, in-depth understanding of how individuals' social world unfolds daily (Brim and Spain, 1974; Pelto, 2013; Cf. Fetterman, 2020).

To gather data, the research team used triangulation. As the term suggests, this approach employs more than one method to collect information (Hobbs and May, 1994; Denzin, 1996; Silverman, 2013; Jerolmack and Khan, 2017). Our research approach involved accessing relevant groups *via* a network of crucial “gatekeepers” (i.e., individuals linked directly to who are active in the studied communities/groups). Based on this negotiated access, the research team gathered data *via* fieldwork from various sources, including direct interviews, observations, and the internet. Moreover, researchers gathered data on the culture, values, and ideology of the participants and groups, and their interactions with each other.

The research team focused on the most active Ultras groups to violence, alleged criminal activity, and the groups' proselytism in and outside the BiH football stadium; the chosen groups needed to represent the three main BiH ethnic groups. *Via* a combination of gatekeepers' introduction and snowballing sampling, we selected the following groups: The HŠK Zrinjski Mostar (Hrvatski športski klub Zrinjski Mostar), the Škripari - NK Široki Brijeg, the Ultras - HŠK Zrinjski Mostar, the Lešinari - FK Borac Banja Luka, and the Robijaši - NK Čelik Zenica.

In relation to the stakeholders, the research team interviewed the following institutional stakeholders:

- The Ministry of Security of BiH;
- The Ministry of Internal Affairs of the Sarajevo Canton;
- The Sarajevo Canton police;
- The Republic of Srpska Ministry of the Interior;
- The Police Administration Banja Luka;
- The Football Association of BiH (Nogometni/Fudbalski Savez Bosne i Hercegovine);
- The Football Association of Republika Srpska (Fudbalski savez Republike Srpske);
- The Football Club Široki Brijeg (Nogometni klub Široki Brijeg);
- The Football Club Željezničar (Fudbalski klub Željezničar Sarajevo);
- The FK Velež Mostar (Fudbalski klub Velež Mostar).

The team then proceeded with semi-structured interviews. Informed consent was given prior to the interviews; the interviews were recorded with a digital recording device, then translated and transcribed by the International Organization for Migration (IOM) is part of the United Nations System administration staff. The observations were carried out in the locations of the cities linked to the groups; those locations were often signposted by graffiti that were also photographed and translated. Observations were also made during matches and events such as the BiH gay pride, where we knew groups would have intervened to protest. To complement this work, the research team gathered and translated media articles, policy documents, and BiH laws dealing with hate crimes and violence at BiH football matches; the research team also collected policies and directives of BiH football clubs and football associations. The institutional stakeholders were instrumental in the collection of these documents. Finally, the online data gathering about groups, networks, and narratives was carried out by the Institute for Strategic Dialogue (ISD); it complemented the offline research by determining the level of online activity of the Ultras groups studied, and if these groups had links to ethno-nationalist violent extremism (c.f. Testa, 2020). The investigation assessed the scale and extent of their online activity, the social media these groups used, how they used them, and for what purposes and the types of content and narratives promoted by the groups.

### Analysis

The research team used grounded theory to make sense of the data (Glaser and Strauss, 1967; Charmaz, 2014). The analysis started by reading and coding^13^ the interviews transcripts, online data, policies, and laws. The team also coded pictures of groups in action and mural graffiti. The codes were at this stage provisional to allow flexibility to new interpretations in line with the development of the analytical process. The process also involved comparing, modifying, and merging codes. Once the research team ended this initial step; the analysis became more intensive, aiming to develop more significant classifications including theoretical concepts; this process was integrated by memo writing. This process lasted until saturation was reached (Strauss and Corbin, 1998). The project adhered to the ethics code of the American Society of Criminology. The online research team also followed The Institute for Strategic Dialogue (ISD's) in-house Ethics Principles for online research. All data obtained for the project was stored securely following the EU General Data Protection Regulation (GDPR).

## Framing the BiH Ultras

Explaining the Ultras in BiH is a complex task. The BiH authorities are struggling to counter the Ultras' illegal activity and control their violence inside and outside the football terraces. The danger posed by these groups is exemplified by a violent incident that occurred in September 2019. Radio Sarajevo came under attack from a group of Ultras associated with the Football Club Sarajevo^14^, terrorising several journalists. Indeed, since early 2019, BiH journalists and the “Free Media Help Line” recorded five death threats and six actual physical assaults on reporters and media teams. The journalists denounced the inability of the BiH authorities to prevent such episodes and to punish them^15^.

The group structure is centralised but includes more than a leader; members also have some authority. So, power is in multiple hands, and there is a high level of functional diversity. In one group, there were ten leaders. The Ultras had a “nucleus” of individuals ranging from 50 to 70, while the group members' number was from 100 to 500 maximum. The age of the members ranged from 17 to a few over 40's years old. The demographic seems to represent the local community's (“the people”) social stratification. The Ultras Zrinjski - FC Zrinjski Mostar provided more details about the demographic of a typical BiH Ultras group:

Research Team: *Who are the members in the group, students or workers?*

Ultras: *There are high-school kids, university students, those*

*who got employed straight after school, or unemployed people - you know how the situation is here* [high unemployment rates].

The leaders were those who were older and were perceived as charismatic figures. This is important to make sense of the radicalisation process of newcomers', as they promote changes in beliefs and behaviours and facilitate the internalisation of the Ultras mentality. The Ultras Lešinari - FC Borac Banja Luka described the nature of the members' commitment to the group:

Research team: *How much time do you invest in the group?*

Ultras: *Usually on weekends, when the football club is in action*.

*We gather maybe every second weekend. However, everyone also leads its own life…*

Research team: *Do you go to every match?*

Ultras: *Every local match, away matches when I have time*.

To be part of the Ultras “nucleus” (senior members' circle), it is indispensable to show commitment and elitism to adhere to the group's values, ideology, and tradition; only, in this case, a member is co-opted. The nucleus deals with all the activities of the Ultras group from memberships, mural graffiti, banners, chanting, smuggling pyrotechnics, and organising crimes such as selling drugs.

## Risk Factors

A range of risk factors explains the existence and appeal of the Ultras groups to the youth of BiH. Our analysis indicates that the first broad risk factor is socio-environmental; therefore, it needs to consider many issues. Ethnicity and ethnic tensions do appear to play a part, but we suggest this is not a prime issue in understanding the Ultras groups in BiH. The Ultras groups exploit ethnicity to justify their existence; the groups serve as a catalyst of belonging in a politically fragmented country. Ethnic roots are also used by the Ultras as a narrative to single out and most often legitimise provocation to rival factions and, ultimately, violence. Our research indicates that the economic and political situation of BiH and unemployment rate and concomitant underutilisation of youth were vital risk factors singled out by all stakeholders, including the Ultras groups. Disenfranchised youth join the Ultras because they allow them to gain power, tackle boredom, discharge everyday frustrations, and because of their productive criminal activities. Our research also suggests that adding to the problems of the political and economic situation are causal factors that relate to the lack of an effective federal legal structure and the fact that the responses of the BiH police and football authorities seem to fall well below international standards; the latter point was confirmed ironically by several Ultras groups.

Data also indicate the BiH football stadiums as a key risk factor, particularly the poor stadium facilities, lack of stadium regulation, and inefficient security. As in other East European countries, low attendances at football matches work as an amplifier of Ultras' actions and presence (Dzhekova et al., 2015 in Testa, 2020, p.28). Ultras are thus perceived by youngsters and other fans as powerful: the true owners of the football stadium. Their chants, banners, symbols, and physical intimidation are used to recruit (fans join them because they are intimidated or fascinated by them) or exclude those who oppose their presence and power. This imbalance of power between Ultras and the “others” within the stadiums must be addressed. The control of the football terraces is so strong that the Ultras determine who has access to them. The Catholic Ultras Škripari - NK Široki Brijeg vetted Muslims who exhibit symbols of their religious identity; according to the group, no women with the niqab could access it.

The Škripari - NK Široki Brijeg explained:

*I have a problem with it* [niqab] *but for another reason. My issue with it is that is not part of the Bosnian Muslim tradition, it is imposed by a foreign culture - the Arabs. A vast majority of Muslims here are moderate, European kind of Muslims. I know for a fact that it bothers Bosniaks even more than Serbs or Croats. I did a lot of research into this because I am interested in it, and I saw that this was not something that was ever part of BiH. This was imposed on them* [BiH Muslims].

In addition, the lack of football clubs' security and regulations inside the stadium means the Ultras groups can exercise a very high level of control in the football stadiums.

The second broad risk factor is political and can be identified in the Ultras' narrative of a perceived corrupt political class that fails the BiH youth and society. Around this narrative, the groups organise and recruit. In this case, the Ultras characterise themselves as the sole “resistance” to the federal and local status quo. The BiH Ultras can be identified as resistance groups from the data gathered. All Ultras groups accomplished three main functions; they were a vehicle of anti-systems sentiments, they function as a means of identity shaping, social solidarity maintenance, offering members and potential recruits with frames to make sense of their lives, frustrations, and grievances; providing recruits and members the illusion of self-efficacy to their grievances. (Cf. Adams and Roscigno, 2005; p.71; Diani, 1992). One of the leaders of the Škripari - FC Široki Brijeg explained their resistance against the system and their struggle with the local authorities:

*We are visible and spreading our messages, but we do it from our stands because we cannot change anything* [outside]. *So, if we have a banner that somebody does not like, our police give us troubles about it. We do not get punished in Sarajevo or anywhere else. This does not happen anywhere else* [in BiH].

All the Ultras groups interviewed manifested their oppositions to local political parties who - according to them - have hijacked any societal arena, including policing; sometimes, the groups acted as political/pressure force to contrast local politics as the earlier quotation of the Škripari - FC Široki Brijeg details.

Far-right ideology was part of two groups' collective identity^16^ but it did not appear to be as sophisticated and strong as other European Ultras, for instance, as the Ultras in Italy, Spain, Greece, and some Eastern European groups such as the Polish and Bulgarians. Significant risk factors are also the sense of belonging/community and identity. For example, being from Grbavica in Sarajevo enshrines an identity upon the everyday teenager of being a Manijac^17^. Local networks (family, friend groups, classmates) also amplify the chances to join an Ultras group. Our data also stress risk factors such as the feeling of victimhood against journalists, the federal state, local politicians, the police, the love for the city and the football club, and the excitement of violence and glory.

### The Ultras Mentality

Throughout the study, all the Ultras interviewed referred to their ways of life as the “Ultras mentality”. The concept of Ultras mentality can be understood using Pierre Bourdieu's concept of Habitus: “*a subjective but not individual system of internalised structures, schemes of perception, conception, and action common to all members of the same group or class”* (Bourdieu, 1977; p.86). The Ultras mentality serves to understand the members' group life perceptions and challenges and ultimately their practises, including violence. These internalised structures and schemes of perception shape the subject's (and groups) shared worldview and their awareness of the social space in which the groups are located (Bourdieu, 1977, p.86, 1998; Bourdieu and Wacquant, 2007). The Ultras mentality is acquired as Bourdieu's Habitus by the practise of being a “true” Ultras, by a permutation of influences such as vicinity or distance from other actors (group members; rivals and the police) and ‘mimeticism (Lizardo, 2009). *Via* mimeticism, intangible principles -such as values- and dispositions are transmitted, and these are employed when similar situations and practises reoccur. Lizardo (2009) clarifies how mimeticism works; it intentionally starts focusing on visually accessible role models – in the case of the Ultras, the Ultras leaders- then, this process of motor-schematic mirroring comes to gain a habitual and implicit cast. This mentality shaping process occurs within the group in the football terraces but also during the meetings outside the stadium. As Boudieu's Habitus, the Ultras mentality has a collective dimension since the members' categories of judgment and action that arise from the group are shared by all those subject to the same social conditions and constraints. Ultras who inhabit the same social space develop a comparable mentality and a comprehension-led mechanism influenced by this mental philtre (Simons and Burt, 2011) sharing hopes, choices, and frustrations.

Based on this study data, the BiH Ultras mentality is shaped around four essential elements, namely groups' values, anti-system attitude, past and tradition, and ethnic nationalism. The fundamental values of the “true” BiH Ultras are loyalty, honour, strength, group's unity, and the celebration of “Balkan” masculinity, essentially represented by backwardness, parochialism, traditionalism (Dumančić and Krolo, 2016), and in the Ultras case, aggressiveness and ultimately violence.

Contact sports are part of this mentality as the Ultras Robijaši - FC Čelik Zenica pointed out:

Research team: *Do you like to fight?*

Ultras: *Yes, yes*.

Research team: *Do you train?*

Ultras: *I play rugby….it is the part of being Ultras* [to be tough]. *I go to the university during the week, and the weekend is for the football games. And you know what happens during the games… You must be prepared [to fight] …. That is also the reason why we go to the football games* [to fight].

The previous quote encapsulates the essence of being a BiH Ultras, reflecting a man showing strength *via* rugby. An Ultras Škripari, who was interviewed, was proud of being a kickboxing expert; his combat skills were used by the group to challenge their opponents; he confirmed being always in the front row if fights against the police and rival groups arose.

The daily frustrations against the federal and local politics are externalised by the groups *via* their anti-system attitude. Their resistance (and disgust) to the perceived corrupt federal state and political class are a common element that unites the groups interviewed. This rage borderline hopeless stance is also present when the BiH football establishment situation is analysed. The Football Association of BiH was considered decadent and costly, spending money on “fancy” buildings in Sarajevo but not investing funds in stadiums' facilities and in promoting BiH youth talent in football.

The police were also often the target of all the Ultras groups' anti-system narratives:

Ultras Škripari - FC Široki Brijeg*: They* [Croatian Democratic Union of BiH- HDZ- and the police*] are all linked, while we are completely unrelated to HDZ. It is the proof that once we start digging into something that should not be looked into (from the political point of view) or rise up against the police, or some club's decisions, immediately we get some fines. So, in these situations the city, the football club and the police all get together to work against us. For example, we make some stupid, small thing at the stadium, and immediately tomorrow 20 group members will be taken to the police station and questioned. So that is why it is impossible to make any change*.

As the quotation underlines, the police in Mostar and the city of Široki Brijeg were seen as being used as a political tool; as a means to punish those who oppose the local political party (the HDZ) deemed as corrupt.

#### Past and Tradition

As mentioned earlier, an evaluation of the BiH Ultras phenomenon concentrating solely on ethnic nationalism does not capture in its entirety extremism in the BiH football terraces. The Ultras Robijaši - NK Čelik Zenica explain*ed: “We cannot speak about ethnicity, because we have members who are Bosniaks, Serbs, Croats and that is why we are specific….. There is no place for nationalism in our group”*. According to the Ultras Robijaši - NK Čelik Zenica, the Ultras from Banja Luka or Mostar are seen the same way as those groups from Sarajevo. Aggro can originate from past rivalries. As in many European Ultras groups, friendships and animosities develop similarly; for example, friendships with other groups tend to be based on respect. All groups, who have the same mentality, benefit from this shared system of values. The premise of the Bedouin Syndrome ostensibly controls the establishment of rivalries and allies: friends of an ally become friends, enemies of an ally become enemies (Bruno, in De Biasi and Marchi, 1998).

Historic rivalries between football clubs are linked to the Ultras mentality and its propensity towards forming a “sacral space^18^.” The football terraces – as the districts and neighbourhoods where the Ultras belong – are deemed sacred; they are a physical and symbolic location, which is autonomous from the stadium and the city. Their violation (sacrilege) by opponents promotes violence and call for “sacrifices”. Rather revealing, in 2016, was the “call to arms” of the Manijaci group of the Sarajevo team FK Željezničar to their rivals the Horde Zla (Evil Horde) to attack and destroy the Fukare^19^ when their football team would have played in Sarajevo. All three Ultras groups were mainly Bosniaks, so ethnicity was not the reason for this episode. Although this episode shows the link between violence and football in BiH, it is essential to point out that football fans' violence cannot be compared in severity and significance to other European countries such as Poland, Russia, Italy, and the UK.

#### Ethnic Nationalism

As mentioned earlier, ethnic nationalism is not enough alone to justify the BiH Ultras' existence. As the Ultras Lešinari - FC Borac Banja Luka argued:

Research team: *Is Serb-nationalism important in your group?*

Ultras A: I do not give a f^***^ *about that. My best man is Croat, my wife is Muslim, my grandmother is Muslim*.

Research team: *But what about the younger generation?*

Ultras A: *They are the same. We love our city and the club*.

Ultras B: *We are the same. There are not only Serbs on the stands*.

*It is about the love for the* [football] *club*.

Ethnicity is used arbitrarily and contradictorily by the groups. In some groups, ethnic nationalism was associated with far-right and far-left ideologies. Ethnicity is symbolically exploited as a tool to distinguish themselves from others (De Vos et al., 2006) justifying at times the groups' existence. An Ultras Zrinjski- HŠK Zrinjski Mostar member underlined the use of what they identified as “Bosniak nationalism”, which was part of the Ultras Manijaci and Horde Zla Sarajevo narratives:

*For example, groups from Sarajevo are right-oriented but they are not fascist, more like Bosniak nationalists. But Velež supporters are officially communists; so, they have a conflict of interest. They cannot put a red star on their flag but also want to be Bosniak. Their “leader”- Tito did not want Bosniaks, Serbs or Croats but he wanted Yugoslavs, and that is their internal conflict. Before the war, they were perceived as a Yugoslav club*.

While ethnic nationalism does not entirely justify the groups' existence, the groups' criminal activities do so. To make sense of the BiH existence, focusing on localised power dynamics and the Ultras groups' criminogenic needs is crucial.

#### Criminal Activities

Our analysis suggests that the Ultras phenomenon in BiH is not so much an issue related to politics or religion (cf. ethnic nationalism and far-right or jihadist ideologies) but more about the groups' criminogenic nature, status, and needs within the political, social, and economic geography of their local setting - their cities. The data indicates that in BiH, Ultras groups exist to make money from criminal activities^20^, mainly *via* drug dealing, racketeering, extortions, intimidations, and “services” offered to local politicians during the electoral period. Our data also highlight that in specific locales, authorities' corruption levels allow the local Ultras groups to gain and exercise their power and control. Crime and corruption were so intrinsically linked to the Ultras in BiH that one Ultras group regretted that it was relatively small in number because this was hindering their criminal opportunities and profits. A key driver for groups' recruitment is their capacity to operate as a semi-organised crime group within and sometimes beyond their locality. Our findings also suggest that those within Ultras groups' organised criminality may be legitimised through the connexions between the Ultras leaders and the football clubs. Our data suggest that most of the Ultras groups receive financial payment from the football clubs to avoid creating problems for their clubs^21^.

While the political and ideological dimensions appear not as significant when compared to their counterparts in Europe and the Balkans^22^, we also found that homophobia hate crimes are instead the element that links all the BiH Ultras groups regardless of religion and ethnicity, and they are an integral part of the Ultras “DNA”. A representative of the Football Association of BiH elaborated on an episode involving homophobia which would have been rigorously dealt with if it had taken place in other football stadiums in Europe:

*Homophobia is present occasionally; during the recent match of FC Željezničar, Manijaci used a banner which said “Ima Zabraniti” (which would mean “there is something to forbid”) as a reaction to the announcement that the first gay pride ever will be held in September 2019 in Sarajevo. The official motto of the pride is “Ima izaći” which means “getting out of the closet” and also getting out to support the pride; additionally, during the same match, the flag of Brunei was displayed as homosexualism there is illegal [punished with the death penalty]*.

Antisemitism shares the same dynamic occurring for homophobic hate crimes; it unites, *via* prejudice and hate all Ultras groups regardless of religion, ethnicity, and historic rivalries.

#### The Internet

Football and social media are closely linked in BiH. For instance, the Bosnian international footballer Edin Dzeko was the most followed page in the country on both Facebook and Twitter, with over 2 million and 1.5 million followers^23^. During this study, the public activity of several Ultras groups was monitored. For the purpose of this analysis public activity is understood to be content produced by particular accounts or Ultras groups on social media which is readily available to researchers searching across a platform, or through that platform's application programming interface (API), and which does not require special permission to access (e.g., through a request to a closed group or chat channel). Hence, the research team focused on engagement data with public Facebook pages associated with Ultras groups in BiH from January 2013 to November 2019. This investigation revealed over 4.7 million user interactions with posts in these groups; 1.5 million (non-unique) users liking these pages and an 11% growth over the 12-month period.

Overall, there was a strong online presence of pages, channels, and accounts associated with BiH Ultras groups. From our data, there were approximately 31,500 core users which follow the Ultras groups and related pages from BiH on Facebook. The findings show that the pages were primarily used for (1) sharing football-related news; (2) mobilising Ultras through glorifying their clubs, stoking rivalries with other groups, and the provision of practical guidance around match days; (3) voicing displeasure with their own club's management or ownership; (4) fundraising and selling merchandise; and (5) spreading political messages. The Ultras' Facebook pages were key in sharing messages and guidance for the group members. Most importantly, the platforms were used to recruit and mobilise individuals to attend home and away games. Each separate Ultras group displayed its rhetoric when mobilising members by using parts of chants glorifying the club and emotional calls to inspire as many individuals to come as possible. The mobilisation calls were not intended for established Ultras members but for a greater audience, primarily individuals from the neighbourhood or city where the club is based and its surroundings. For instance, looking at the mobilisation called by the Ultras Fukare from Tuzla calls upon all attendance to “one of the most important games this season” away at Ugljevik, the FK Zvijezda 09 stadium. The call was for all “fans, sympathisers, old and young” to come in large numbers to support the team, so they do not relive the relegation suffered by the club in 2012 after 42 years in the top tier Bosnian football”.

Ethnic nationalist narratives and political ideologies were present in their posts. Events from the Yugoslav wars are consistently present in the Ultras' rhetoric; the groups commemorate various massacres and atrocities that happened during the war. Inevitably, each group commemorates certain events based on their ethnic identity. For example, the Alcohol Boys from Prijedor, a Bosnian Serb Ultras group, commemorate Operation Storm (Oluja), which saw the dislocation of ethnic Serbs from the Srpska Krajina region in Croatia in 1995. Similarly, the Škripari - FC Široki Brijeg - and their rival Ultras Zrinjski- FC Zrinjski Mostar, ethnic Croat Ultras groups, commemorate the fall of Vukovar when the Yugoslav National Army (JNA) took over the city in a Pyrrhic victory from the Croatian forces that left thousands of civilians dead, injured and displaced. This event is seen as the liberation of Vukovar by some Serb groups.

## Concluding Thoughts

This article originates from a research project aimed to increase the key BiH stakeholders' understanding of the relations between extreme football fans groups (Ultras) and violent extremism. The research project contributed towards IOM-UN and USAID's effort to reduce the threat of ideologically motivated violence in BiH. This article argues that the BiH Ultras are an issue that must be thoughtfully and systematically addressed because of their associations with criminal activity and how their behaviours in football stadiums enable their power and influence to enact crime (including hate crimes). This article highlights the importance of an efficient response in terms of policing, club security arrangements, and new legislation^23^.

## Data Availability Statement

The raw data supporting the conclusions of this article will be made available by the authors, without undue reservation.

## Ethics Statement

Ethical review and approval was not required for the study on human participants in accordance with the local legislation and institutional requirements. Written informed consent for participation was not required for this study in accordance with the national legislation and the institutional requirements.

## Author Contributions

The author confirms being the sole contributor of this work and has approved it for publication.

## Funding

This project was funded by the United States Agency for International Development and the BiH Resilience Initiative IOM – The UN Migration Agency.

## Conflict of Interest

The author declares that the research was conducted in the absence of any commercial or financial relationships that could be construed as a potential conflict of interest.

## Publisher's Note

All claims expressed in this article are solely those of the authors and do not necessarily represent those of their affiliated organizations, or those of the publisher, the editors and the reviewers. Any product that may be evaluated in this article, or claim that may be made by its manufacturer, is not guaranteed or endorsed by the publisher.

## Footnotes

^2^2022 Data, retrieved February 2022 from the World Population Review : https://worldpopulationreview.com/countries/bosnia-and-herzegovina-population

^3^C.f. https://balkaninsight.com/2020/12/10/bosnia-charges-serb-chetniks-with-inciting-ethnic-hatred/

^4^C.f. https://balkaninsight.com/2017/05/05/far-right-balkan-groups-flourish-on-the-net-05-03-2017/

^5^C.f. https://balkaninsight.com/2021/06/02/bosnian-far-right-movement-weds-bosniak-nationalism-neo-nazism/

^6^The main group is known as the “Maniacs” or Manijaci, they are linked to Bosniaks and are based in Sarajevo.

^7^Retrieved November 2020 from https://balkaninsight.com/2017/05/05/far-right-balkan-groups-flourish-on-the-net-05-03-2017/ and https://www.iss.europa.eu/sites/default/files/EUISSFiles/Brief%2020%20Balkan%20foreign%20fighters.pdf

^8^Retrieved September 2020 from https://css.ethz.ch/en/services/digital-library/articles/article.html/108850/pdf; https://balkaninsight.com/2015/03/16/hooligan-attack-raises-ethnic-tensions-in-bosnia/

^9^Retrieved November 2020 from https://balkaninsight.com/2015/03/16/hooligan-attack-raises-ethnic-tensions-in-bosnia/

^10^This also holds true for mainstream extremism, especially jihadist and far-right groups.

^11^Fielding (1981) in his seminal study on the British National Front, details the challenges for a researcher in studying extremist groups objectively; while Testa (2010a, 2010b), Testa, 2018, 2020) points out the risk inherent in interacting with them.

^12^There are differences between academic ethnography and applied ethnography. While the theoretical roots are the same, the main difference is in how the research is shaped. In academic ethnography, the chosen topic/problem dictates the design, budget, and, most importantly, the timeframe; specifically, fieldwork requires six to two years or more. A contract funds the applied ethnographic work, and it is a fully developed response to the funders' expressed interest in the problem (Brim and Spain, 1974; Pelto, 2013; Cf. Fetterman, 2020). The timeframe of the fieldwork depends on the funders' needs, and the findings are used to tackle the problem.

^13^All data were coded and analysed using MAXQDA software, which facilitates and supports qualitative, quantitative and mixed methods research projects (Woolf and Silver, 2018).

^14^In terms of criminal activities, the Maniacs (Manijaci supporters of the Football Club Željezničar) and Horde Zla (supporters of the FC Sarajevo) are deemed by all police forces interviewed as the most dangerous; they are classified as criminal organisations. It is important to stress that both Ultras groups were the only ones who refused at the last moment to meet the research team because of our questions focusing on criminal activities.

^15^Retrieved September 2020 from https://balkaninsight.com/2019/09/30/bosnia-journalists-protest-after-thugs-storm-news-outlet

^16^Škripari - NK Široki Brijeg and Ultras - HŠK Zrinjski Mostar.

^17^Ultras supporting the Football Club Željezničar (Sarajevo).

^18^C.f. http://rj-vko.kz/en/speczproektyi/sakralnaya-geografiya.html

^19^They are called the “Wretches” and they are the Ultras of the football team Sloboda from the city of Tuzla.

^20^This was confirmed by the BiH authorities.

^21^This was confirmed by the BiH authorities.

^22^The Ministry of Security of BiH, Sarajevo Canton police, Republic of Srpska Ministry of the Interior and Police Administration Banja Luka confirmed our data that racism was not an issue.

^23^Data gathered by the Institute for Strategic Dialogue for this research (2020).
